# The effect of e-health interventions on meeting the needs of individuals with infertility: a narrative review

**DOI:** 10.1186/s43043-023-00137-7

**Published:** 2023-04-28

**Authors:** Azam Hamidzadeh, Shahrbanoo Salehin, Tahereh Naseri Boori Abadi, Reza Chaman, Naser Mogharabian, Afsaneh Keramat

**Affiliations:** 1 Student Research Committee, School of Nursing and Midwifery, Shahroud University of Medical Sciences, Shahroud, Iran; 2 Reproductive Studies and Women’s Health Research Center, Shahroud University of Medical Sciences, Shahroud, Iran; 3 Department of Health Information Technology, School of Allied Medical Sciences, Shahroud University of Medical Sciences, Shahroud, Iran; 4 Center for Health-Related Social and Behavioral Sciences Research, Shahroud University of Medical Sciences, Shahroud, Iran; 5 Sexual Health and Fertility Research Center, Shahroud University of Medical Sciences, Shahroud, Iran; 6 School of Nursing and Midwifery, Shahroud University of Medical Sciences, Shahroud, Iran

**Keywords:** Infertility, Internet, Web, E-health, RCT

## Abstract

**Background:**

The mental health and well-being of millions of people worldwide are negatively impacted by infertility. A promising solution to meet the needs of people suffering from infertility is e-health interventions, such as online counseling and support groups. This study aims to review the current literature on e-health interventions and how they impact people with infertility.

**Main body of the abstract:**

Relevant studies were searched in PubMed, Web of Science, and Scopus databases. Articles were entered into the EndNote software and screened for duplicates and relevance. Two authors then reviewed full-text articles independently, with a third person resolving any disagreements. Thirteen studies conducted between 2007 and 2022 were identified. The interventions aimed to meet various needs, including training on drug use (*n* = 23), lifestyle modifications (*n* = 1), periconceptional behavior modifications (*n* = 1), drug management (*n* = 1), IVF training (*n* = 4), psychological support to reduce distress (*n* = 4), and promoting a positive sexual self-concept (*n* = 1).

**Short conclusion:**

The limited number of e-health interventions for infertile patients, the heterogeneity of interventions, and the lack of long-term effectiveness data make it challenging to compare e-health interventions to nonelectronic alternatives. However, the increasing use of technology in healthcare, especially during and after the Covid-19 pandemic, suggests that e-health educational interventions such as those using the Internet, psychological support, and patient interaction will continue to play a crucial role in healthcare.

**Supplementary Information:**

The online version contains supplementary material available at 10.1186/s43043-023-00137-7.

## Background

Infertility is a complex health issue that affects both physical and emotional well-being. According to WHO, female infertility is the 5th most severe disability globally [1]. Infertile couples face numerous challenges such as sexual dysfunction, financial strain due to the high costs of treatment, and psychological distress including depression, anxiety, and social stigma. These individuals may also face domestic violence and feelings of failure [2].

Infertility can impact individuals in various ways, affecting both men and women [3]. Meeting their multidimensional training needs is crucial to address their specific requirements and enhancing their overall well-being [4]. Identifying their psychosocial and counseling needs is necessary to design effective interventions that can provide them with the required support [5].

Fortunately, there are a variety of training options, both electronic and non-electronic, to fulfill the needs of patients struggling with infertility [6, 7]

Various nonelectronic interventions are available for those struggling with infertility, such as body-mind group interventions, counseling, supportive interventions, psychosocial interventions, and therapeutic interventions aimed at changing multiple lifestyle behaviors [8]. However, it is essential to note that further research with proper methods is needed to establish conclusive evidence [9].

The emergence of electronic health interventions has genuinely transformed how we provide health services. With the advent of telemedicine, mobile applications, Internet-based systems, and computerized medical information, patients can easily access quality care from the comfort of their homes [10]. Interactive technologies such as personal digital assistants, interactive televisions, interactive voice response systems, computer kiosks, and mobile communications have revolutionized how patients interact with healthcare providers. These advancements are crucial in enhancing healthcare services, ultimately leading to better patient outcomes [11].

Much research has been conducted on e-health interventions for infertility. These interventions incorporate strategies to alleviate stress, anxiety, and depression while offering guidance on empowering infertile couples through lifestyle adjustments, dietary habits, social support, sexual self-concept, and promoting infertility-related awareness.

As the importance of remote access technologies continues to grow, particularly amid the Covid-19 pandemic, we must design e-health interventions that cater to the diverse needs of our society. In this regard, we aim to shed light on the research field of e-health interventions in the context of infertility.

## Methods

### Search strategy and selection criteria

**Fig. 1:**
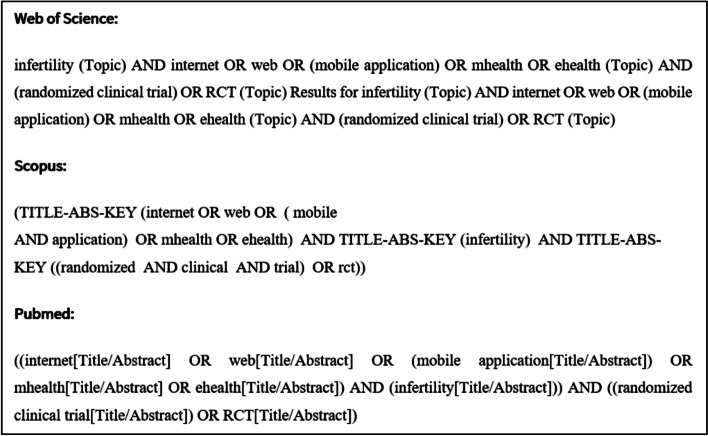
Search strategy in main databases

We conducted a thorough search on articles related to “Internet” and “e-health” in combination with “infertility,” “IVF,” and “reproductive techniques assisted” and their synonyms using citation databases such as Web of Science, Scopus, and PubMed (Fig. 1).”

The inclusion criteria included studies that used information and communication technologies, such as the Internet, web, smartphone applications, and e-health, to meet the needs of infertile patients. Studies that conducted nonelectronic and electronic interventions on individuals other than infertile patients and articles not published in English were excluded from the study.

### Data extraction

Data was gathered and organized data from various sources, including citation databases and manual searches. Employing EndNote software, we meticulously sifted through the articles and eliminated duplicates before screening the title and abstract. The full text of each article was then independently reviewed by two authors, and any disagreements were resolved with the supervision of a third party. Our data extraction table was carefully compiled, detailing the author’s name, year of publication, study location, type of intervention, the sample size in the intervention and control groups, and the findings of each study.

## Results

**Fig. 2:**
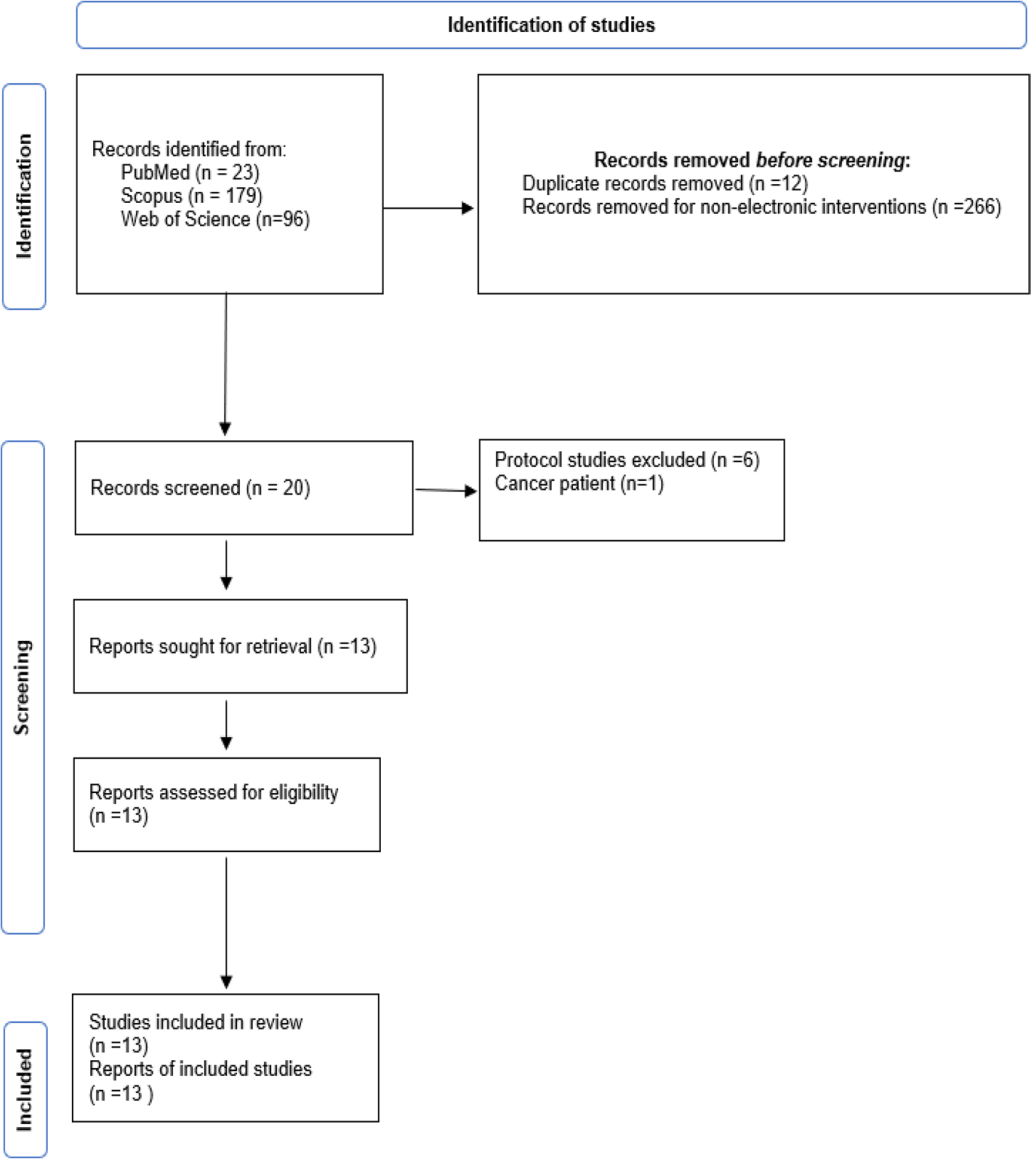
Flow diagram of study selection

We thoroughly searched and screened 298 articles from the Web of Science, Scopus, and PubMed databases. After removing duplications and irrelevant literature, we selected 13 relevant studies conducted between 2007 and 2022 that aligned with our inclusion criteria. These studies investigated the effectiveness of e-health interventions in addressing the needs of infertile patients (Fig. 2).

Most studies regarding on e-health interventions have primarily been conducted in the USA (5 studies) and the Netherlands (4 studies), with additional studies in Iran, Switzerland, England, and Canada (1 study each). Of the 13 studies, 4 utilized mobile health interventions [12-14], and 9 employed Internet-based interventions [15-23].

The current interventions cater to the needs of infertile patients by providing training on the proper use of medication [23], promoting modifications in nutrition and lifestyle [13], encouraging changes in periconceptional behaviors [12], managing drug use [18], offering IVF-related training [16, 20, 22, 24], addressing psychological distress [16, 17, 19, 21], and promoting positive sexual self-concept [14]. Out of the 13 reviewed studies, 10 targeted infertile women [12, 14-16, 18, 19, 21-24], while only three included couples with infertility [13, 17, 20].

Tables 1 and 2 showcase the different types and characteristics of e-health interventions designed to cater to the needs of infertile patients.

**Table 1:** Types of e-health interventions to meet the needs of individuals with infertility

**Author, year, place**	**Target population**	**Sample size**	**Type of intervention**	**Control**	**Meeting the need**	**The purpose of the study**
Adeleye et al., 2022, USA [23]	Women with infertility	**257**	Online educational video on the preparation and administration of drug during ovulation stimulation	Educational video about IVF	Correct use of fertility drugs	• Evaluating the effectiveness of the educational videos on increasing self-efficacy, fertility treatment, quality of life, and reducing perceived stress • Evaluating the effectiveness of educational videos on increasing confidence in the treatment and reducing medication errors
NG et al., 2021, England [12]	Women with infertility	**262**	Online smartphone application	Website of standard lifestyle recommendations for the women planning to become pregnant	Periconceptional behavior	Evaluating the effectiveness of online lifestyle coaching platform Smarter Pregnancy in modifying periconceptional behaviors
Martin et al., 2021, USA [18]	Women with infertility	**153**	OnTrack web-based application	Traditional medication management	Medication management during the treatment	Evaluating the use of a web-based application in facilitating medication management during IVF treatment
Timmers et al., 2021, Netherlands [24]	Women with infertility	**54**	Patient Journey app + website and brochure	Website and brochure	Training the different stages of IVF	• Evaluating the capacity of app to support patients undergoing IVF treatment in all stages of treatment • Evaluating the effectiveness of the application
Clifton et al., 2020, USA [15]	Women with infertility	**71**	Mind/body program for fertility	Waiting-list	Mind/body for infertile individuals	• Evaluating the effect of program on the willingness of participants to participate in the intervention • Evaluating the reduced distress during the intervention period and increased probability of getting pregnant
Oostingh et al., 2020, Netherlands [13]	Men and women with infertility undergoing IVF/ICSI treatment	848	The regular version of mobile health program	The light version of the mobile health program	Nutrition and lifestyle	Examining the compliance and effectiveness of mHealth nutrition and lifestyle coaching program Smarter Pregnancy in couples undergoing in vitro fertilization (IVF) treatment with or without intracytoplasmic sperm injection
Yazdani et al, 2019, Iran [14]	Women with infertility	**80**	Online counseling via social networks on smartphone	Routine infertility counseling	Sexual self-concept	Evaluating the effect of sexual counseling through social networks on a smartphone in the sexual self-concept of infertile women
Vause et al., 2017, Canada [22]	Women with infertility	40	Interactive web-based teaching tool to introduce IVF	Traditional nurse-led teaching sessions	• Knowledge about IVF • Perceived stress • Satisfaction	Comparing the effectiveness of web-based teaching tools with the traditional learning by nurses in the patients undergoing IVF treatment
Van Dongen et al., 2016, Holland [21]	Women with infertility	**120**	digiCOACH personalized Internet program (psychoeducational and cognitive behavioral therapy digital program — including three modules: stress management, depressed mood, and social support)	Traditional care	Improved request for support	Evaluating a personalized e-therapy program (Internet based) for women undergoing assisted reproductive technologies to reduce clinically relevant symptoms of anxiety or depression after unsuccessful treatment
Sexton et al., 2010, USA [19]	Women with infertility	31	Web-based treatment (translate elements of a written bibliotherapy package to an interactive web-based format)	Waiting-list	Treatment of infertility-related psychological distress	Evaluating the effectiveness of the psychological intervention on reducing general and infertility-related stress in women undergoing medical treatment to solve fertility problems
Haemmerli et al., 2010, Switzerland [17]	Men and women with infertility	124	Child Wish Online coaching program	Waiting-list	The need for information and interaction with the therapist	Evaluating the effectiveness of the program to reducing depression and anxiety levels of clinically distressed and depressed participants
Cousineau et al., 2008, USA [16]	Women with infertility	190	Online psychoeducational support program	-	Psychoeducational support	Evaluating the effectiveness of an online psychoeducational support program for women with infertility
Tuil et al., 2007, Holland [20]	Men and women with the infertility undergoing IVF/ICSI treatment	180	Online access to information related to the infertility and IVF treatment, access to medical records, and communication with doctors through e-mail, form, discussion, and chat room		General and specific information related to treatment and facilities for doctors and patients	Evaluating the effect of Internet-based personal health records on the empowerment of patients undergoing IVF

**Table 2:** Characteristics of e-health interventions to meet the needs of infertile patients

Author, year, place	Type of RCT	The evaluated parameters	Important findings	Conclusion
Adeleye et al., 2022, USA [23]	Randomized, double-blinded, placebo-controlled trial	• Self-efficacy • Stress or treatment burden	• No significant difference between the two groups due to self-efficacy, fertility quality of life treatment, and perceived stress using an intention-to-treat analysis • Increasing the confidence of the intervention group in medication administration for treatment by 4 times (*OR* 4.70 and *p* < 0.01) • The reduction of medication errors in the intervention group (*OR* 0.37 and *p* = 0.03)	Educational videos about fertility drugs have no effect on psychological health but improve confidence in the treatment and reduce medication errors
NG et al., 2021, England [12]	Prospective RCT iPLAN trial	• Nutrition risk score at 12 weeks • Lifestyle risk score at 12 weeks • Adherence to the program at 12 and 24 weeks after randomization	• Reduction of composite risk score at the 12th week in the intervention group (composite risk score = risk score for intake of folic acid, vegetables and fruits, tobacco, and alcohol use) • The difference in CRS between the intervention and control groups was −0.47 (95% confidence interval −0.97 to 0.02) at 12 weeks and −0.32 (95% confidence interval: −0.82 to 0.15) at 24 weeks • Significant reduction of lifestyle risk scores in women with a body mass index of 25 kg/m^2^ or above compared to women with a body mass index of below than 25 kg/m^2^ • The chance of getting pregnant at the 24th weeks in the intervention group increased compared to the control group (*OR*: 2.83, 95% *CI*: 0.35 to 57.76)	The online lifestyle coaching platform is more effective to provide the lifestyle advice and modifying behaviors to support women with a history of infertility or repeated miscarriages
Martin et al., 2021, USA [18]	Multicenter RCT	• Residual drug • Occurrence of medication errors • The amount of sent messages and patient -phone calls • Patient satisfaction	• No significant difference between the two groups due to the average number of portal messages (*p* = 0.532) and telephone calls (*p* = 0.394), estimated number of remaining units of rFSH (*p* = 0.829), HMG vials (*p* = 0.329), or the doses of GnRH antagonist (*p* = 0.947), the number of errors in medication administration (*p* = 0.339), 12 patients in the control group (69/12, 14.4%) and 8 patients in the intervention group (8/72, 11.1%) ○ No significant difference between the two groups due to the amount of the remaining drug, the estimated cost of drug waste in the control group (US $2578 ± US $2056) and in the case group (US $2554 ± US $1855) • No significant difference between the two groups due to patients’ satisfaction with the clinic experience (*p* = 0.916)	The OnTrack application did not affect reducing medication errors, medication surplus, or the number of messages sent from the patient. This application and others like it can improve patient-doctor communication during IVF treatment, increase satisfaction, and more successful pregnancy Many patients had medication surplus, which is necessary to pay attention to reduce costs during IVF
Timmers et al., 2021, Netherland [24]	RCT	• The satisfaction level with the information provided in the application • Level of knowledge • Ability to take medication • Quality of treatment • Quality of healthcare • Application usage	• Increased satisfaction with the information received 2 days after the IVF (*p* = .004) • Increased level of knowledge 2 days after IVF consultation in the intervention group (*p* < .001)	Compared to standard training, using an application to provide timely information about IVF treatment increases the patient satisfaction Using this application increases people’s knowledge about steps and procedures of IVF treatment, and finally, the usage statistics of this application show the information needs of the patients and their willingness to use an e-health application as part of treatment
Clifton et al., 2020, USA [15]	A randomized pilot trial	• Retention • Adhere • Satisfaction • Distress • Pregnancy	○ To be comparable to the retention, adherence, and satisfaction rates to those reported in other Internet-based RCTs ○ Significant reduction in distress (anxiety, *p* = .003; depression, *p* = .007; stress, *p* = .041 fertility social, *p* = .018; fertility sexual, *p* = .006), estimated as medium-to large effect sizes (*ds* = 0.45 to 0.86) ○ Higher odds of becoming pregnant 4.47 times for intervention group participants as compared to wait-list group, *OR*, 95% *CI* [1.56, 12.85], and *p* = .005 and occurred earlier	Smarter Pregnancy Coaching platform improves the consumption of vegetables, fruits, and folic acid supplements and reduces smoking and alcohol consumption in couples undergoing IVF/ICSI
Oostingh et al., 2020, Netherlands [13]	Multicenter, single-blinded, randomized controlled trial	Modification of inappropriate nutritional behaviors based on reducing the DRS at 24 weeks after the program started Modification of nutritional behaviors, and lifestyle at 36 weeks after the program started	• Significant improvement in the nutritional behaviors of women and men in the intervention group compared to the control group after 24 weeks of coaching (*Ɓ* = 0.779, 95% confidence interval [CI] 0.456–1.090 for women; *Ɓ* = 0.826, 95% *CI* 0.416–1.284 for men) after 24 weeks of coaching • Significant improvement of inadequate lifestyle behaviors of women (*Ɓ* = 0.108, 95% *CI* 0.021–0.203)	The Smarter Pregnancy mHealth coaching program improves crucial habits for IVF/ICSI couples
Yazdani and colleagues, 2019, Iran [14]	Parallel RCT	Sexual self-concept	○ Significant differences between two groups during the time in terms of positive self-concept domain (120.4 ± 17.9 versus 105.1 ± 16.8) • An increasing trend of the scores in positive sexual self-concept domain (before intervention 110.6 ± 18.42), (after intervention 120.1 ± 18.7), and (4 weeks after intervention 120.4 ± 17.9) (*p* < 0.001) • Decrease in negative sexual self-concept domain (before intervention 24.3 ± 7.87), (after intervention 20.2 ± 7.77), and (4 weeks after intervention 19.65 ± 6.97) (*p* < 0.001) • No difference between two groups in terms of situational self-concept (*p* = 0.06)	Counseling via social networks improves the sexual self-concept and sexual relations of infertile couples
Vause et al., 2017, Canada [22]	Prospective RCT	• Knowledge regarding the IVF process, risks, and logistics assessed before and after the training session • Patient stress before and after the training session • Patient satisfaction after the training session and embryo transfer day	○ Increase in knowledge score in two groups after training (p < 0.001) ○ Decrease in stress score in two groups after training (*p* = 0.05) • Increasing satisfaction of intervention group with web-based educational tools (p = 0.01) ○ Increase in knowledge score in two groups after training (*p* < 0.01) ○ Decrease in stress score in two groups after training (*p* = 0.54) • Increasing satisfaction of intervention group with web-based educational tools (*p* = 0.01) ○ Increasing in knowledge score in both groups after the intervention • No significant difference between participants of high- versus low-income and education status due to knowledge (*p* = 0.21), stress (*p* = 0.912), and satisfaction (*p* = 0.24)	This study’s findings showed that web-based educational tools have a similar effect on increasing knowledge and reducing stress compared to traditional education in both groups One of the advantages of web-based training is the greater satisfaction of patients undergoing IVF treatment
Van-Dongen et al., 2016, Holland	Single-center, 2-arm, parallel-group, single-blind feasibility RCT	• Demand • Acceptability • Usability • Integration implementation	○ Acceptability of e-treatment program due to current clinical and care guidelines ○ Due to a lack of feeling of need for help, only 44% of people participate in this program (demand) ○ Dropout rate relatively high causes average assessment of applicability ○ Clearly, this intervention is effective (decrease in the percentage of women with clinical symptoms related to the anxiety or depression in the intervention group compared to control group 3 months after the first ART cycle risk difference of 24% (*p* = 0.03)	Personalized e-therapy program in clinical fertility care to the risk profile of patients is promising and feasible
Sexton et al., 2010, USA [19]	RCT	• General stress • Stress infertility	Reduction of general stress in the intervention group (after interactive web-based bibliotherapy) (*p* = .048)	The general stress of infertile women is significantly reduced using an online cognitive behavioral approach
Haemmerli et al., 2010, Switzerland [17]	RCT	• Depression • Anxiety • Contact • Distress • Infertility • Fertility rate	• Decreasing in the level of depression in the intervention group 5 months after the intervention compared to before the intervention (*p* = 0.007) • Reducing the general level of anxiety in the intervention group 5 months after the intervention compared to before the intervention (*p* < 0.01) • Reducing state-trait anxiety level in the intervention group 5 months after the intervention compared to before the intervention (*p* < 0.01)	Most of the participants (80%) rated Internet support as positive or very positive, and based on high demand for such support, Internet-based interventions are a new and promising approach for infertile patients and need more development and evaluation
Cousineau et al., 2008, USA [16]	RCT	• Infertility distress • Self-efficacy of infertility • Decisional conflict • Marital cohesion • Coping style • The dosage of the program and satisfaction with the program 1 month after the use of the program	Enhancing the performance of women participating in the online group program in the following areas: • Social concerns related to infertility distress (*p* = 0.038) • Feeling more informed about medical decisions with which they were contending (*p* = 0.037) • Reducing general stress (*p* = 0.10) • Reducing sexual concerns (*p* = 0.059) • Reducing distress related to life without children (*p* = 0.063) • Increasing Infertility self-efficacy (*p* = 0.067) • Increasing decision-making clarity (*p* = 0.079) In the group that women used, an online program for more than 1 h is as follows: • Reducing general stress (*p* = 0.028) • Increasing self-efficacy (*p* = 0.024)	This evidence-based e-health program showed that web-based educational intervention has beneficial effects on various psychological domains and is a useful resource for fertility practices
Tuil et al., 2007, Holland [20]	RCT	Patients’ empowerment in the following: • Participation in decision-making • General and specific self-efficacy • Actual and perceived knowledge • Side effects • Satisfaction • Helplessness • Acceptance • Social support • State anxiety • Depression	• No significant differences were observed in per-person change in patient empowerment (*p* > 0.05) • No significant differences regarding per-person change in patient satisfaction (*p* > 0.05) • No significant differences in the per-person changes of variables related to infertility problems, social support, anxiety, and depression (*p* > 0.05)	Using the personalized health records did not affect patient empowerment and did not have an adverse effect on people

## Discussion

We utilized RCT designs in the studies reviewed. Our pioneering analysis of clinical trials explores the effectiveness of e-health interventions for subfertile patients.

The selection of studies with RCT design was based on their position at the top of the evidence hierarchy and their frequent use in designing interventions and enhancing clinical care. To improve clinical practices, it is crucial to know the evidence supporting effective interventions. Therefore, conducting further evaluations and research can be advantageous. Applying validated research findings in clinical practice can promote interventions and cultivate practical guidelines [25]. As professionals, we understand the significance of reliable evidence and its role in improving clinical care. e-Health interventions in reproductive health consist of supportive, educational, and mental health-promoting interventions delivered through several multimedia or interactive modules [26]. Our study categorizes interventions into two groups based on their ability to address the needs of individuals experiencing infertility.

The first group consists of interventions designed to meet the educational needs of affected individuals, focusing on increasing knowledge about infertility and its treatments while correcting inappropriate nutritional and lifestyle behaviors. The second group focuses on providing emotional and psychological support to infertility patients. This is in line with e-health-related review studies in other fields of medicine [27, 28].

In their review study, Aarts et al. (2012) identified patient-centered Internet interventions for infertility care, which they categorized and evaluated through a detailed synthesis. Results showed that Internet-based interventions could effectively provide support and education and promote mental health in this field. By incorporating interactive and dynamic components, interventions are successfully designed for optimal outcomes. Methodological standards are also emphasized to ensure complex interventions are conducted and evaluated accurately. Overall, patient-centered Internet interventions can greatly improve outcomes in infertility treatment [26].

E-health interventions have the capability to revolutionize reproductive medicine by focusing on critical factors such as exercise, diet, and lifestyle choices. These interventions can greatly enhance the success rates of assisted reproductive technology [29].

As per recent studies, lifestyle changes implemented before or during infertility treatment can effectively enhance therapeutic outcomes. A meta-analysis study on lifestyle interventions for polycystic ovary syndrome patients revealed that such interventions could improve metabolic parameters like weight loss and insulin resistance, positively impacting infertility treatment. These findings highlight the importance of lifestyle changes in the realm of reproductive health [30].

Interventions aimed at lifestyle changes that help improve fertility may be particularly promising and beneficial when delivered via the Internet [31].

Van Dijk et al. demonstrated that personalized mHealth coaching can effectively empower both fertile and infertile couples to adopt healthy lifestyle changes, improve nutrition, and boost their chances of conception [32].

As healthcare professionals, we recognize the importance of lifestyle modifications in addressing infertility. However, we understand that implementing these changes can be challenging due to high drop-out rates and a lack of continuous participation by patients [33].

To overcome this obstacle, Internet-based interventions may prove to be a promising solution by providing patients with access and maintaining continuity of care. Ultimately, incorporating these interventions into the treatment plan can positively impact the pregnancy rate of infertile patients [34].

Kim et al. (2018) examined the characteristics and effectiveness of online interventions for infertile women and mentioned there is evidence of the effectiveness of online intervention for this group. These interventions increase the probability of pregnancy and reduce the level of stress. As barriers to traditional individual and couples counseling, including stigma, financial problems, fear, and commute challenges, can affect the reception of service, new technologies such as mobile apps and Internet-based programs can be a proper and practical option to reduce mental problems [35].

According to Simionescu et al. (2021), infertility can heighten feelings of stress and anxiety, negatively affecting fertility. To improve the chances of successful treatment, managing stress is crucial. Online interventions aimed at reducing stress can positively impact the treatment process [36].

In a study conducted by Safdari et al., the effectiveness of mobile health interventions in enhancing the mental and emotional well-being of patients with infertility was explored. The authors revealed that utilizing a self-care application to interact between patients and medical staff during infertility treatment decreased patient anxiety [37].

“Infotility” is an e-health intervention designed to cater to infertility patients’ psychoeducational and psychosocial needs. This application provides reliable information on infertility’s medical and psychosocial dimensions and treatment options. This platform hosts a peer support forum, which has effectively reduced stress and empowered patients [38].

As numerous online interventions are available for stress reduction and psychological support in infertility, comparing their effectiveness can be daunting. Determining their ability to address stress and other psychological factors related to infertility can be challenging. Hence, a comprehensive analysis is required [28].

Infertility and its treatment can significantly impact the lives of couples, causing disruptions in their marital and sexual relationships [39].

Lotfollahi et al. found that infertile women scored lower in sexual satisfaction, control by others, and fear of intercourse than fertile women. They highlighted the importance of reproductive health professionals in empowering women’s sexual self-concept through educational and counseling interventions [40].

It is essential to consider the challenges couples face in discussing sexual issues. Providing counseling sessions in a comfortable setting is crucial to their success. Internet- and mobile-based psychological interventions seem to be a valuable addition to routine care, empowering individuals to promote their sexual health on a guided self-help basis [41].

The study conducted by Yazdani et al. highlights the effectiveness of utilizing smartphones and social media to offer sexual counseling, incorporating audio and text files. This approach has positively impacted individuals’ self-concept and promoted healthier sexual relationships between partners [14].

Despite widespread usage of social media worldwide, only 51 publications have been identified by Gabarron (2016) regarding promoting sexual health through social media. Encouragingly, around 25% of these studies showed positive results, suggesting that social media interventions can positively impact sexual health. Nevertheless, further research is necessary to establish a solid evidence base for the field. This research must focus on the theoretical framework and employ robust research designs to further validate these findings [42].

As we have observed, there remains a lack of research into the impact of e-health interventions on treatment expenses [18]. However, it is important to note that investing in e-health has the potential to result in decreased direct care costs, improved accessibility to health services, and ultimately better health outcomes - particularly for nations with limited resources. This is a promising prospect that should not be overlooked [43].

As individuals who are experiencing infertility, it is crucial to fully comprehend the proper usage and potential side effects of the medications prescribed.

The results of our study indicate that patients with infertility can greatly benefit from watching educational videos regarding the proper use of medication. By doing so, patients become more aware and confident in administering their medication, resulting in a fourfold increase in medication administration confidence and a decrease in medication errors. Our findings suggest that incorporating educational videos into patient care can play a crucial role in improving patient outcomes [23].

Our review has shown that all studies on e-health interventions were conducted in countries with middle to high income. This unequal allocation implies the potential for global disparities in the development and implementation of such interventions. Thus, experts and researchers should prioritize the creation of a suitable platform that enables the adoption and execution of evidence-based and culturally sensitive digital interventions in countries where digital health is still emerging.

To successfully implement e-health interventions, it is crucial to consider the accessibility of the Internet to all citizens. Additionally, access to digital devices and services is necessary for digital interventions. However, the costs of promoting Internet interventions in non-Western countries with limited resources can pose significant challenges. Therefore, it is imperative for future research in digital health to address these challenges and promote the sustainable and secure development of health services in low-resource countries.

The Internet is a valuable resource for accessing information, but its reliability remains a concern. In particular, when it comes to making health-related decisions, the potential impact of unreliable online data cannot be overstated. Uncertainty surrounding the accuracy of this information can have serious consequences for individuals’ health. As such, it is important to address this issue and ensure that citizens have access to high-quality, trustworthy health information online [44].

As professionals, we understand that individuals facing fertility disorders often seek information online. However, Sexton et al. warn that online resources may not provide reliable mental healthcare information and should not be solely relied upon [19]. Limited health knowledge and web literacy can lead to misinterpretations of online medical data, which may account for these disparities. The reliability of web resources depends on several factors, including the source of information, timeliness, quality of research, peer review, and accessibility. By considering these factors, individuals can determine which web resources are reliable and which ones are not, which can help them increase their knowledge levels effectively [45].

Implementing a suitable training program and assessment tools is strongly recommended to enhance users’ abilities and encourage a meticulous approach to evaluating health information online. This approach would promote more excellent proficiency and accuracy in assessing health-related content.

## Conclusion

Looking at our review, it is evident that e-health interventions have shown promising results by alleviating stress, anxiety, and depression, fostering sound knowledge regarding infertility and its treatment, nurturing better lifestyle and nutrition habits, and enhancing sexual satisfaction for women struggling with infertility.

The proliferation of information and communication technology, particularly in the wake of the Covid-19 pandemic, necessitates the development and implementation of e-health interventions through the Internet or mobile devices. As such, healthcare professionals should design educational interventions, provide emotional support, and encourage patient interaction to meet the needs of individuals and couples seeking medical care in this digital age.

### Limitation

It is important to note that the study has limitations. Firstly, there is a lack of e-health interventions available for individuals and couples struggling with infertility. Additionally, there are variations in the interventions offered and insufficient evidence of their long-term effects.

Therefore, it was challenging to compare the effectiveness of e-health interventions with nonelectronic ones. Due to these factors, the results of this study should be approached with caution. Furthermore, extensive research is necessary to obtain a more precise understanding of the effectiveness of e-health interventions for infertility.

## Supplementary Information

**Additional file 1.** The effect of e-health interventions on meeting the needs of individuals with infertility: a narrative review.

## Data Availability

The datasets used and/or analyzed during the current study are available from the corresponding author on reasonable request.
