# Supplementary material for: The effect of e-health interventions on meeting the needs of individuals with infertility: a narrative review
Source: Middle East Fertil Soc J. 2023 Apr 28;28(1):12. doi: 10.1186/s43043-023-00137-7 (PMC10140700; doi:10.1186/s43043-023-00137-7)
Supplement: Supplementary file 1 — Additional file 1. The effect of e-health interventions on meeting the needs of individuals with infertility: a narrative review. [file 43043_2023_137_MOESM1_ESM.docx]

**The effect of e-health interventions on meeting the needs of individuals with infertility: a narrative review**

**Abstract**

Background: The mental health and well-being of millions of people worldwide are negatively impacted by infertility. A promising solution to meet the needs of people suffering from infertility is e-health interventions, such as online counseling and support groups. This study aims to review the current literature on e-health interventions and how they impact people with infertility.

Main body of the abstract: : Relevant studies were searched in the PubMed, Web of Science, and Scopus databases. Articles were entered into Endnote software and screened for duplicates and relevance. Two authors then reviewed full-text articles independently, with a third person resolving any disagreements.Thirteen studies conducted between 2007 and 2022 were identified. The interventions aimed to meet various needs, including training on drug use (n=23), lifestyle modifications (n=1), periconceptional behavior modifications (n=1), drug management (n=1), IVF training (n=4), psychological support to reduce distress (n=4), and promoting a positive sexual self-concept (n=1).

Short Conclusion: The limited number of e-health interventions for infertile patients, the heterogeneity of interventions, and the lack of long-term effectiveness data make it challenging to compare e-health interventions to non-electronic alternatives. However, the increasing use of technology in healthcare, especially during and after the Covid-19 pandemic, suggests that e-health educational interventions such as those using the internet, psychological support, and patient interaction will continue to play a crucial role in healthcare.

Keywords: Infertility, Internet, Web, eHealth, RCT

**Introduction**

Infertility is a complex health issue that affects both physical and emotional well-being. According to WHO, female infertility is the 5th most severe disability globally (1). Infertile couples face numerous challenges such as sexual dysfunction, financial strain due to the high costs of treatment, and psychological distress including depression, anxiety, and social stigma. These individuals may also face domestic violence and feelings of failure (2).

Infertility can impact individuals in various ways, affecting both men and women. (3). Meeting their multidimensional training needs is crucial to address their specific requirements and enhancing their overall well-being (4). Identifying their psychosocial and counseling needs is necessary to design effective interventions that can provide them with the required support (5) .

Fortunately,there are a variety of training options, both electronic and non-electronic, to fulfill the needs of patients struggling with infertility (6, 7).

Various non-electronic interventions are available for those struggling with infertility, such as body-mind group interventions, counseling, supportive interventions, psychosocial interventions, and therapeutic interventions aimed at changing multiple lifestyle behaviors (8). However, it is essential to note that further research with proper methods is needed to establish conclusive evidence (9).

The emergence of electronic health interventions has genuinely transformed how we provide health services. With the advent of telemedicine, mobile applications, internet-based systems, and computerized medical information, patients can easily access quality care from the comfort of their homes(10). Interactive technologies such as personal digital assistants, interactive televisions, interactive voice response systems, computer kiosks, and mobile communications have revolutionized how patients interact with healthcare providers. These advancements are crucial in enhancing healthcare services, ultimately leading to better patient outcomes (11).

Much research has been conducted on eHealth interventions for infertility. These interventions incorporate strategies to alleviate stress, anxiety, and depression while offering guidance on empowering infertile couples through lifestyle adjustments, dietary habits, social support, sexual self-concept, and promoting infertility-related awareness.

As the importance of remote access technologies continues to grow, particularly amid the Covid - 19 pandemic, we must design e-health interventions that cater to the diverse needs of our society. In this regard, we aim to shed light on the research field of e-health interventions in the context of infertility.

Methods

Search strategy and selection criteria: We conducted a thorough search on articles related to 'Internet' and 'eHealth' in combination with 'Infertility', 'IVF' and 'Reproductive techniques assisted' and their synonyms using citation databases such as Web of Science, Scopus, and PubMed (Fig. 1).

The inclusion criteria included studies that used information and communication technologies, such as the Internet, web, smartphone applications, and e-health, to meet the needs of infertile patients. Studies that conducted non-electronic and electronic interventions on individuals other than infertile patients and a**rticles not published in English were excluded from the study.**

Data extraction: Data was gathered and organized data from various sources, including citation databases and manual searches. Employing Endnote software, we meticulously sifted through the articles and eliminated duplicates before screening the title and abstract. The full text of each article was then independently reviewed by two authors, and any disagreements were resolved with the supervision of a third party. Our data extraction table was carefully compiled, detailing the author's name, year of publication, study location, type of intervention, the sample size in the intervention and control groups, as well as the findings of each study.

**Results**

We thoroughly searched and screened 298 articles from the Web of Science, Scopus, and PubMed databases. After removing duplications and irrelevant literature, we selected 13 relevant studies conducted between 2007 and 2022 that aligned with our inclusion criteria. These studies investigated the effectiveness of e-health interventions in addressing the needs of infertile patients. **Figure 2 showcases the study selection procedure.**

Most studies regarding on e-health interventions have primarily been conducted in the United States (5 studies) and the Netherlands (4 studies), with additional studies in Iran, Switzerland, England, and Canada (1 study each). Of the 13 studies, 4 utilized mobile health interventions (12-14) and 9 employed internet-based interventions (15-23).

The current interventions cater to the needs of infertile patients by providing training on the proper use of medication (23), promoting modifications in nutrition and lifestyle (13), encouraging changes in periconceptional behaviors (12), managing drug use (18), offering IVF-related training (16, 20, 22, 24), addressing psychological distress (16, 17, 19, 21), and promoting positive sexual self-concept (14). Out of the 13 reviewed studies, 10 targeted infertile women(12, 14-16, 18, 19, 21-24), while only three included couples with infertility (13, 17, 20).

**Tables 1 and 2 showcase the different types and characteristics of e-health interventions designed to cater to the needs of infertile patients.**

**Discussion:**

We utilized RCT designs in the studies reviewed. Our pioneering analysis of clinical trials explores the effectiveness of e-health interventions for subfertile patients.

The selection of studies with RCT design was based on their position at the top of the evidence hierarchy and their frequent use in designing interventions and enhancing clinical care. To improve clinical practices, it's crucial to know the evidence supporting effective interventions. Therefore, conducting further evaluations and research can be advantageous. Applying validated research findings in clinical practice can promote interventions and cultivate practical guidelines(25).As professionals, we understand the significance of reliable evidence and its role in improving clinical care

e-Health interventions in reproductive health consist of supportive, educational, and mental health-promoting interventions delivered through several multimedia or interactive modules(26). Our study categorizes interventions into two groups based on their ability to address the needs of individuals experiencing infertility.

The first group consists of interventions designed to meet the educational needs of affected individuals, focusing on increasing knowledge about infertility and its treatments while correcting inappropriate nutritional and lifestyle behaviors. The second group focuses on providing emotional and psychological support to infertility patients. This is in line with eHealth-related review studies in other fields of medicine(27, 28).

In their review study, Aarts et al. (2012) identified patient-centered internet interventions for infertility care, which they categorized and evaluated through a detailed synthesis. Results showed that Internet-based interventions could effectively provide support and education and promote mental health in this field. By incorporating interactive and dynamic components, interventions are successfully designed for optimal outcomes. Methodological standards are also emphasized to ensure complex interventions are conducted and evaluated accurately. Overall, patient-centered internet interventions can greatly improve outcomes in infertility treatment(26)

E-health interventions have the capability to revolutionize reproductive medicine by focusing on critical factors such as exercise, diet, and lifestyle choices. These interventions can greatly enhance the success rates of assisted reproductive technology(29).

As per recent studies, lifestyle changes implemented before or during infertility treatment can effectively enhance therapeutic outcomes. A meta-analysis study on lifestyle interventions for polycystic ovary syndrome patients revealed that such interventions could improve metabolic parameters like weight loss and insulin resistance, positively impacting infertility treatment.These findings highlight the importance of lifestyle changes in the realm of reproductive health(30).

Interventions aimed at lifestyle changes that help improve fertility may be particularly promising and beneficial when delivered via the Internet(31).

Van Dijk et al. demonstrated that personalized mHealth coaching can effectively empower both fertile and infertile couples to adopt healthy lifestyle changes, improve nutrition, and boost their chances of conception(32).

As healthcare professionals, we recognize the importance of lifestyle modifications in addressing infertility. However, we understand that implementing these changes can be challenging due to high drop-out rates and a lack of continuous participation by patients(33).

To overcome this obstacle, internet-based interventions may prove to be a promising solution by providing patients with access and maintaining continuity of care. Ultimately, incorporating these interventions into the treatment plan can positively impact the pregnancy rate of infertile patients(34).

Kim et al. (2018) examined the characteristics and effectiveness of online interventions for infertile women and mentioned there is evidence of the effectiveness of online intervention for this group. These interventions increase the probability of pregnancy and reduce the level of stress. As barriers to traditional individual and couples counseling, including stigma, financial problems, fear, and commute challenges, can affect the reception of service, new technologies such as mobile apps and Internet-based programs can be a proper and practical option to reduce mental problems(35).

According to Simionescu et al. (2021), infertility can heighten feelings of stress and anxiety, negatively affecting fertility. To improve the chances of successful treatment, managing stress is crucial. Online interventions aimed at reducing stress can positively impact the treatment process(36).

In a study conducted by Safdari et al., the effectiveness of mobile health interventions in enhancing the mental and emotional well-being of patients with infertility was explored. The authors revealed that utilizing a self-care application to interact between patients and medical staff during infertility treatment decreased patient anxiety(37).

Infotility" is an e-health intervention designed to cater to infertility patients' psycho-educational and psychosocial needs. This application provides reliable information on infertility's medical and psychosocial dimensions and treatment options. This platform hosts a peer support forum, which has effectively reduced stress and empowered patients(38).

As numerous online interventions are available for stress reduction and psychological support in infertility, comparing their effectiveness can be daunting. Determining their ability to address stress and other psychological factors related to infertility can be challenging. Hence, a comprehensive analysis is required(28)

Infertility and its treatment can significantly impact the lives of couples, causing disruptions in their marital and sexual relationships(39).

Lotfollahi et al. found that infertile women scored lower in sexual satisfaction, control by others, and fear of intercourse than fertile women. They highlighted the importance of reproductive health professionals in empowering women's sexual self-concept through educational and counseling interventions(40).

It is essential to consider the challenges couples face in discussing sexual issues. Providing counseling sessions in a comfortable setting is crucial to their success. Internet- and mobile-based psychological interventions seem to be a valuable addition to routine care, empowering individuals to promote their sexual health on a guided self-help basis(41).

The study conducted by Yazdani et al. highlights the effectiveness of utilizing smartphones and social media to offer sexual counseling, incorporating audio and text files. This approach has positively impacted individuals' self-concept and promoted healthier sexual relationships between partners(14).

Despite widespread usage of social media worldwide, only 51 publications have been identified by Gabarron (2016) regarding promoting sexual health through social media. Encouragingly, around 25% of these studies showed positive results, suggesting that social media interventions can positively impact sexual health. Nevertheless, further research is necessary to establish a solid evidence base for the field. This research must focus on the theoretical framework and employ robust research designs to further validate these findings(42).

As we have observed, there remains a lack of research into the impact of e-health interventions on treatment expenses(18). However, it is important to note that investing in e-health has the potential to result in decreased direct care costs, improved accessibility to health services, and ultimately better health outcomes - particularly for nations with limited resources. This is a promising prospect that should not be overlooked(43).

As individuals who are experiencing infertility, it is crucial to fully comprehend the proper usage and potential side effects of the medications prescribed.

The results of our study indicate that patients with infertility can greatly benefit from watching educational videos regarding the proper use of medication. By doing so, patients become more aware and confident in administering their medication, resulting in a four-fold increase in medication administration confidence and a decrease in medication errors. Our findings suggest that incorporating educational videos into patient care can play a crucial role in improving patient outcomes(23).

Our review has shown that all studies on eHealth interventions were conducted in countries with middle to high income. This unequal allocation implies the potential for global disparities in the development and implementation of such interventions. Thus, experts and researchers should prioritize the creation of a suitable platform that enables the adoption and execution of evidence-based and culturally sensitive digital interventions in countries where digital health is still emerging.

To successfully implement e-health interventions, it is crucial to consider the accessibility of the internet to all citizens. Additionally, access to digital devices and services is necessary for digital interventions. However, the costs of promoting internet interventions in non-western countries with limited resources can pose significant challenges. Therefore, it is imperative for future research in digital health to address these challenges and promote the sustainable and secure development of health services in low-resource countries.

The Internet is a valuable resource for accessing information, but its reliability remains a concern. In particular, when it comes to making health-related decisions, the potential impact of unreliable online data cannot be overstated. Uncertainty surrounding the accuracy of this information can have serious consequences for individuals' health. As such, it's important to address this issue and ensure that citizens have access to high-quality, trustworthy health information online(44).

As professionals, we understand that individuals facing fertility disorders often seek information online. However, Sexton et al. warn that online resources may not provide reliable mental healthcare information and should not be solely relied upon (19). Limited health knowledge and web literacy can lead to misinterpretations of online medical data, which may account for these disparities. The reliability of web resources depends on several factors, including the source of information, timeliness, quality of research, peer review, and accessibility. By considering these factors, individuals can determine which web resources are reliable and which ones are not, which can help them increase their knowledge levels effectively(45).

Implementing a suitable training program and assessment tools is strongly recommended to enhance users' abilities and encourage a meticulous approach to evaluating health information online. This approach would promote more excellent proficiency and accuracy in assessing health-related content.

**Conclusion**

Looking at our review, it's evident that e-health interventions have shown promising results by alleviating stress, anxiety, and depression, fostering sound knowledge regarding infertility and its treatment, nurturing better lifestyle and nutrition habits, and enhancing sexual satisfaction for women struggling with infertility.

The proliferation of information and communication technology, particularly in the wake of the COVID-19 pandemic, necessitates the development and implementation of e-health interventions through the internet or mobile devices. As such, healthcare professionals should design educational interventions, provide emotional support, and encourage patient interaction to meet the needs of individuals and couples seeking medical care in this digital age.

**Limitation**

It is important to note that the study has limitations. Firstly, there is a lack of e-health interventions available for individuals and couples struggling with infertility. Additionally, there are variations in the interventions offered and insufficient evidence of their long-term effects.

Therefore, it was challenging to compare the effectiveness of e-health interventions with non-electronic ones. Due to these factors, the results of this study should be approached with caution. Further, extensive research is necessary to obtain a more precise understanding of the effectiveness of e-health interventions for infertility.

**Ethics Approval**

This study was approved by the Ethics Committee of Shahroud University of Medical Sciences (IR.SHE.REC.1400.087

**Acknowledgements**

The authors express their sincere gratitude to the Research Deputy of Shahroud University of Medical Sciences. We are greatly appreciative of their support.

**Conflict of interest**

The authors declare no conflict of interest.

**Refrences**:

1. Akhondi MM, Kamali K, Ranjbar F, Shirzad M, Shafeghati S, Ardakani ZB, et al ( 2013). Prevalence of primary infertility in Iran in 2010. Iranian journal of public health,42(12):1398. Available at: http://ijph.tums.ac.ir

2. Maharlouei N, Morshed Behbahani B, Doryanizadeh L, Kazemi M (2021).Prevalence and pattern of infertility in Iran: A systematic review and meta-analysis study. Women’s Health Bulletin.,8(2):63-71.  <https://doi.org/10.30476/whb.2021.89924.1102>

3. Hajizade-Valokolaee M, Khani S, Fooladi E, Peivandi S, Habibi F, Rahmani Z(2018).Investigating sexual and reproductive health and its related factors in infertile women. Journal of Mazandaran University of Medical Sciences.,27(158):154-65. URL: <http://jmums.mazums.ac.ir/article-1-10724-en.html>

4. Mikkelsen AT, Madsen SA, Humaidan P (2013). Psychological aspects of male fertility treatment. Journal of advanced nursing,69(9):1977-86. <https://doi.org/10.1111/jan.12058>

5. Sylvest R, Fürbringer JK, Schmidt L, Pinborg A (2016). Infertile men’s needs and assessment of fertility care. Upsala journal of medical sciences,121(4):276-82. https://doi.org/10.1080/03009734.2016.1204393

6. Hesari ZHNA, Lotfi R, Pouragha B, Badehnoosh B, Yazdkhasti M (2019). The need for a training software among iranian infertile couples: a qualitative study. International Journal of Fertility & Sterility..13(2):118.  doi: [10.22074/ijfs.2019.5727](https://doi.org/10.22074%2Fijfs.2019.5727)

7. Ezabadi Z, Mollaahmadi F, Sazvar S, Vesali S, Omani-Samani R (2019

).Satisfaction with information provided to infertile patients who undergo assisted reproductive treatment. International journal of fertility & sterility,12(4):324. doi: [10.22074/ijfs.2019.5314](https://doi.org/10.22074%2Fijfs.2019.5314)

8. Polillo A, Gran-Ruaz S, Sylvestre J, Kerman N (2021).The use of eHealth interventions among persons experiencing homelessness: A systematic review. Digital health.,7:2055207620987066. doi: [10.1177/2055207620987066](https://doi.org/10.1177%2F2055207620987066)

9. Stevenson JK, Campbell ZC, Webster AC, Chow CK, Tong A, Craig JC, et al (2019).eHealth interventions for people with chronic kidney disease. Cochrane Database of Systematic Reviews ,(8). doi: [10.1002/14651858.CD012379.pub2](https://doi.org/10.1002/14651858.cd012379.pub2)

10. Ng KYB, Steegers-Theunissen R, Willemsen S, Wellstead S, Cheong Y, Macklon N (2021).Smartphone-based lifestyle coaching modifies behaviours in women with subfertility or recurrent miscarriage: a randomized controlled trial. Reproductive BioMedicine Online.,43(1):111-9. doi: 10.1016/j.rbmo.2021.04.003

11. van Buul AR, Kasteleyn MJ, Arends JM, Shi T, Kelly DP, Chavannes NH, et al ( 2020). eHealth only interventions and blended interventions to support self-management in adolescents with asthma: A systematic review. Clinical eHealth,3:49-62. doi:[10.1016/j.ceh.2020.06.001](http://dx.doi.org/10.1016/j.ceh.2020.06.001)

12. Oostingh EC, Koster MP, van Dijk MR, Willemsen SP, Broekmans FJ, Hoek A, et al(2020).First effective mHealth nutrition and lifestyle coaching program for subfertile couples undergoing in vitro fertilization treatment: a single-blinded multicenter randomized controlled trial. Fertility and sterility,114(5):945-54. doi: 10.1016/j.fertnstert.2020.04.051.

13. Timmers T, Keijsers M, Kremer JA, Janssen L, Smeenk J (2021).Supporting Women Undergoing IVF Treatment With Timely Patient Information Through an App: Randomized Controlled Trial. JMIR mHealth and uHealth, 9(8):e28104. doi: 10.2196/28104.

14. Yazdani M, Mahmoodi Z, Azin SA, Qorbani M (2019 ).The effect of counseling based on sexual self-concept via social networks on smartphone in infertile women: a randomized controlled trial. International Journal of Community Based Nursing and Midwifery .7(3):231. doi: [10.30476/IJCBNM.2019.44998](https://doi.org/10.30476%2FIJCBNM.2019.44998)

15. Clifton J, Parent J, Seehuus M, Worrall G, Forehand R, Domar A (2020).An internet-based mind/body intervention to mitigate distress in women experiencing infertility: a randomized pilot trial. PLoS One,15(3):e0229379. doi: [10.1371/journal.pone.0229379](https://doi.org/10.1371%2Fjournal.pone.0229379)

16. Cousineau TM, Green TC, Corsini E, Seibring A, Showstack MT, Applegarth L, et al (2008).Online psychoeducational support for infertile women: a randomized controlled trial. Human Reproduction, 23(3):554-66. doi: 10.1093/humrep/dem306.

17. Hämmerli K, Znoj H, Berger T (2010).Internet-based support for infertile patients: a randomized controlled study. Journal of behavioral medicine, 33:135-46. doi: 10.1007/s10865-009-9243-2.

18. Martin CE, Lanham M, Almgren-Bell A, Marsh C, Omurtag K (2021).A randomized controlled trial to evaluate the use of a web-based application to manage medications during in vitro fertilization. Fertility and Sterility,116(3):793-800. doi: 10.1016/j.fertnstert.2021.04.022

19. Sexton MB, Byrd MR, O’Donohue WT, Jacobs NN (2010).Web-based treatment for infertility-related psychological distress. Archives of Women's Mental Health.,13:347-58. doi: 10.1007/s00737-009-0142-x.

20. Tuil WS, Verhaak CM, Braat DD, de Vries Robbé PF, Kremer JA (2007). Empowering patients undergoing in vitro fertilization by providing Internet access to medical data. Fertility and sterility,88(2):361-8. doi: 10.1016/j.fertnstert.2006.11.197.

21. van Dongen AJ, Nelen WL, IntHout J, Kremer JA, Verhaak CM (2016). e-Therapy to reduce emotional distress in women undergoing assisted reproductive technology (ART): a feasibility randomized controlled trial. Human Reproduction, 31(5):1046-57. doi: 10.1093/humrep/dew040.

22. Vause TDR, Allison DJ, Vause T, Tekok-Kilic A, Ditor DS, Min JK (2018) .Comparison of a web-based teaching tool and traditional didactic learning for in vitro fertilization patients: a preliminary randomized controlled trial. Journal of Obstetrics and Gynaecology Canada,40(5):588-94. doi: 10.1016/j.jogc.2017.08.029

23. Adeleye A, Cruz K, Cedars MI, Pasch L, Huddleston H (2022). Learning from Online Video Education (LOVE) improves confidence in fertility treatments: a randomized controlled trial. NPJ Digital Medicine. 5(1):128. https://doi.org/10.1038/s41746-022-00673-y

24. Aarts J, Van den Haak P, Nelen W, Tuil W, Faber M, Kremer J (2012).Patient-focused internet interventions in reproductive medicine: a scoping review. Human reproduction update,18(2):211-27. doi: 10.1093/humupd/dmr045

25. Haagen EC, Tuil W, Hendriks J, Bruijn Rd, Braat DD, Kremer JA (2003).Current Internet use and preferences of IVF and ICSI patients. Human reproduction ,18(10):2073-8. doi: 10.1093/humrep/deg423.

26. Van Dijk MR, Huijgen NA, Willemsen SP, Laven JS, Steegers EA, Steegers-Theunissen RP (2016). Impact of an mHealth platform for pregnancy on nutrition and lifestyle of the reproductive population: a survey. JMIR mHealth and uHealth,4(2):e5197. doi: 10.2196/mhealth.5197.

27. van Dijk MR, Koster MP, Willemsen SP, Huijgen NA, Laven JS, Steegers-Theunissen RP (2017). Healthy preconception nutrition and lifestyle using personalized mobile health coaching is associated with enhanced pregnancy chance. Reproductive biomedicine online.;35(4):453-60.  doi: 10.1016/j.rbmo.2017.06.014

28. Miner SA, Gelgoot EN, Lahuec A, Wunderlich S, Safo D, Brochu F, et al (2022).Who needs an app? Fertility patients’ use of a novel mobile health app. Digital Health., 8:20552076221102248. doi: [10.1177/20552076221102248](https://doi.org/10.1177%2F20552076221102248)

29. Emokpae MA, Brown SI (2021).Effects of lifestyle factors on fertility: practical recommendations for modification. Reproduction & Fertility, 2(1):R13.  doi: [10.1530/RAF-20-0046](https://doi.org/10.1530%2FRAF-20-0046)

30. Langarizadeh M, Fatemi Aghda SA, Nadjarzadeh A (2022). Design and evaluation of a mobile-based nutrition education application for infertile women in Iran. BMC Medical Informatics and Decision Making, 22(1):58. https://doi.org/10.1186/s12911-022-01793.

31. Moghadam AD, Delpisheh A, Sayehmiri K (2014).The trend of infertility in Iran, an original review and meta-analysis. Nursing Practice Today, 1(1):46-52. Available online at: http://npt.tums.ac.ir or https://www.researchgate.net/publication/303225019

32. Safdari R, Choobineh H, Sedaghatzadeh M (2019) .The Design of Mobile-Based Self-Care Application Program for Infertility Treatment Using Assisted Reproductive Technology (ART). Payavard Salamat ,13(4):311-23. URL: <http://payavard.tums.ac.ir/article-1-6866-en.html>

33. Kruglova K, O'Connell SBL, Dawadi S, Gelgoot EN, Miner SA, Robins S, et al (2021). An mHealth app to support fertility patients navigating the world of infertility (Infotility): Development and usability study. JMIR Formative Research, 5(10):e28136.  doi: [10.2196/28136](https://doi.org/10.2196/28136)

34. Lotfollahi H, Riazi H, Omani-Samani R, Maroufizadeh S, Montazeri A (2021). Sexual self-concept in fertile and infertile women: a comparative study. International Journal of Fertility & Sterility ,15(1):60. doi: 10.22074/ijfs.2021.6205
